# Functions and clinical significance of KLRG1 in the development of lung adenocarcinoma and immunotherapy

**DOI:** 10.1186/s12885-021-08510-3

**Published:** 2021-06-29

**Authors:** Xiaodong Yang, Yuexin Zheng, Zhihai Han, Xiliang Zhang

**Affiliations:** 1 Department of General Surgery, The Sixth Medical Center of PLA General Hospital, No. 6 Fucheng Road, Haidian District, Beijing, 100048 China; 2 Department of Pulmonary and Critical Care Medicine, The Sixth Medical Center of PLA General Hospital, Beijing, China

**Keywords:** KLRG1, Lung adenocarcinoma, Prognosis, Immunotherapy

## Abstract

**Background:**

As a marker of differentiation, Killer cell lectin like receptor G1 (KLRG1) plays an inhibitory role in human NK cells and T cells. However, its clinical role remains inexplicit. This work intended to investigate the predictive ability of KLRG1 on the efficacy of immune-checkpoint inhibitor in the treatment of lung adenocarcinoma (LUAD), as well as contribute to the possible molecular mechanisms of KLRG1 on LUAD development.

**Methods:**

Using data from the Gene Expression Omnibus, the Cancer Genome Atlas and the Genotype-Tissue Expression, we compared the expression of KLRG1 and its related genes Bruton tyrosine kinase (BTK), C-C motif chemokine receptor 2 (CCR2), Scm polycomb group protein like 4 (SCML4) in LUAD and normal lung tissues. We also established stable LUAD cell lines with KLRG1 gene knockdown and investigated the effect of KLRG1 knockdown on tumor cell proliferation. We further studied the prognostic value of the four factors in terms of overall survival (OS) in LUAD. Using data from the Gene Expression Omnibus, we further investigated the expression of KLRG1 in the patients with different responses after immunotherapy.

**Results:**

The expression of KLRG1, BTK, CCR2 and SCML4 was significantly downregulated in LUAD tissues compared to normal controls. Knockdown of KLRG1 promoted the proliferation of A549 and H1299 tumor cells. And low expression of these four factors was associated with unfavorable overall survival in patients with LUAD. Furthermore, low expression of KLRG1 also correlated with poor responses to immunotherapy in LUAD patients.

**Conclusion:**

Based on these findings, we inferred that KLRG1 had significant correlation with immunotherapy response. Meanwhile, KLRG1, BTK, CCR2 and SCML4 might serve as valuable prognostic biomarkers in LUAD.

**Supplementary Information:**

The online version contains supplementary material available at 10.1186/s12885-021-08510-3.

## Key points

KLRG1 inhibited the progress of LUAD.

The expression of KLRG1 had correlation with immunotherapy response.

KLRG1, BTK, CCR2 and SCML4 might serve as valuable prognostic biomarkers in LUAD.

## Background

Lung cancer is the principal cause of cancer deaths worldwide [1, 2]. Most patients have advanced disease when they are diagnosed with lung cancer. Patients in early-stage can receive surgical, chemo- or radiation therapy, but over 90% of the patients will unavoidably have disease recurrence. The overall 5-year survival rate is lower than 60% [2, 3].

Non-small cell lung cancers (NSCLCs), account for 85% of lung tumors, include a variety of cancer types, such as squamous cell cancers (LUSCs), adenocarcinomas (LUADs), and large cell lung cancers. Among them, LUSCs and LUADs are the largest NSCLC subgroups. Meanwhile, lung adenocarcinoma (LUAD) is the most heterogeneous and aggressive among all NSCLC subtypes. LUAD is the most common type of lung cancers among nonsmokers. The incidence of LUAD is higher among women than men, and it is more likely to happen in younger people than other types of lung cancer. In the past few decades, LUAD has replaced LUSC as the most frequent histological subtype [4].

LUADs originate from cells that secrete surfactant ingredients. The most important morphological features of LUADs include acinar, solid, papillary, micropapillary, and invasive mucinous types. At the same time, a small part of LUADs shows colloid, enteric or fetal features. The staining of thyroid transcription factor 1 (TTF-1/NKX2–1) or napsin-A (NAPSA) can be used to support the diagnosis when the morphological feature of adenocarcinoma is unclear. The sensitivity of the two markers is approximately 80% for the identification of LUAD [5].

Although chemotherapy, radiotherapy, targeted therapy and immunotherapy have made huge progress in the past decade, the prevention, early detection and treatment of LUAD are still facing great challenges. More research is needed to understand the molecular mechanisms facilitating the development of lung carcinogenesis.

The clinical development of immune-checkpoint inhibitors has created an exhilarant era of anticancer therapies. Durable responses have been seen in patients with lung cancer, melanoma and other malignancies [6, 7]. Pembrolizumab, an anti-PD-1 antibody, in combination with pemetrexed, was approved by European Medicines Agency (EMA) as first-line treatment of metastatic LUAD. Atezolizumab, an anti-PD-L1 antibody, in combination with carboplatin/paclitaxel/bevacizumab was granted Food and Drug Administration (FDA) approval in untreated LUAD patients. Although monotherapy with anti-PD-1 or PD-L1 drugs is usually well tolerated, the combination treatment increases the risk of immune-related adverse events [8]. So, biomarkers with predictive role need to be developed to augment patient benefit, diminish the risk of toxicity, and guide the combination approaches [9, 10]. Although the expression of PD-L1 on tumor cells positivity improves the clinical benefit population, PD-L1 detection alone is not satisfactory for patient selection and efficacy prediction in most malignancies [11]. The investigation of powerful biomarkers to help predict the response and clinical benefit of immune-checkpoint inhibitors is crucial to further advance the field of precision immunotherapy [12, 13].

In this study, through the analysis of the database and clinical samples, we found four markers (KLRG1, BTK, CCR2 and SCML4) which may play important roles in the development of lung adenocarcinoma. Knockdown of KLRG1 promoted the proliferation of A549 and H1299 lung tumor cells. Additionally, the expression of KLRG1 is positively correlated with the efficacy of immune-checkpoint inhibitors. Collectively, KLRG1 may be a powerful biomarker to support the diagnosis and predict the clinical benefit of immunotherapy in lung adenocarcinoma.

## Methods

### Extraction and analysis of data from the TCGA database

The raw data of RNAseqv2 in 518 LUAD cases were downloaded from The Cancer Genome Atla (TCGA) database. Their clinicopathological information, including age at initial pathologic diagnosis, smoking history, gender, nodal status, pathologic stage, residual tumors, recurrence status, relapse-free survival (RFS) in days, overall survival (OS) status, and OS in days was downloaded. GEPIA2 (http://gepia2.cancer-pku.cn) [14] and Xena Browser (http://xena.ucsc.edu/) [15], two online interactive web server for analyzing the RNA sequencing data of tumors and normal samples from the TCGA and The Genotype-Tissue Expression (GTEx) projects, were used to analyze the expression profiles and prognostic value of selected genes. And the log_2_(TPM + 1) was used the show the expression value of the selected genes.

### Prediction of related genes

The co-expression analysis module of cBioPortal [16, 17] is able to extract genes that are co-expressed with KLRG1 in the database of Lung Adenocarcinoma (TCGA, PanCancer Atlas). The mRNA expression, RSEM (Batch normalized from Illumina HiSeq_RNASeqV2) was used in the co-expression analysis. The associated genes that were identified by cBioPortal were subjected to follow-up pathway analysis.

### Cell line

The A549 human lung adenocarcinoma and H1299 human lung carcinoma cell lines were obtained from the American Type Culture Collection. Cells were maintained in Dulbecco’s modified Eagle’s medium (DMEM) basic medium supplemented with 10% fetal bovine serum and 1% antibiotics at 37 °C with 5% CO_2_.

### Establishment of stable KLRG1 knockdown cells

HEK-293 T cells in T175 flasks were transfected with the packaging vector psPAX2 (9.4 μg), envelope plasmid pVSVG (9.4 μg), and transfer plasmid (18.8 μg) containing the shRNA targeting KLRG1. Following 72 h, the HEK-293 T medium containing the virus was collected and concentrated, and then transferred to A549 and H1299 plate (5 × 10^6^ cells). The medium was replaced after 72 h and cells containing the integrated virus were selected with puromycin (50 ng/μl). The knockdown efficiency were validated by RT-PCR and Western blotting. Sequences of used shRNAs were as follow: KLRG1-shRNA-1: 5′- CCGGGATTGGTCTGAGGAACAATTCCTCGAGGAATTGTTCCTCAGACCAATCTTTTTTG-3′, KLRG1-shRNA-2: 5′- CCGGGATCTGTCATGTATCCCTAAACTCGAGTTTAGGGATACATGACAGATCTTTTTTG-3′, scrambled-shRNA: 5′- CCGGGCGCGATAGCGCTAATAATTTCTCGAGAAATTATTAGCGCTATCGCGCTTTTTTG-3′.

### Western blotting

The knockdown and control A549 or H1299 cells (1 × 10^6^ cells per well) were plated in 12-well plates and cultured for 24 h, then cellular KLRG1 protein levels were evaluated in total cell extracts by Western blot analysis. Blots were developed using enhanced chemiluminescence (ECL) and detected by exposure to chemiluminescence-sensitive film. Antibodies against KLRG1 (rabbit polyclonal), β-actin (mouse monoclonal, AC-15) were from Sigma-Aldrich (St. Louis, MO, USA). The protein bands without overexposure were quantified relative to β-actin expression using ImageJ software (NIH, Bethesda, MD, USA). We obtain the absolute intensity (AI) for each experimental band of KLRG1. Relative intensity (RI) for each experimental band was calculated by normalizing the experimental AI to the corresponding loading control (β-actin) AI.

### RNA extraction and qRT-PCR

Total RNAs were extracted with TRIzol reagent (Invitrogen, Carlsbad, CA, USA), reverse transcribed with PrimeScrip RT-PCR Kit (Takara Biotechnology Co., Ltd., Dalian, China), followed by qRT-PCR with SYBR Premix Ex Taq (Takara Biotechnology Co., Ltd., Dalian, China). QuantStudio 5 real-time PCR system (Applied Biosystems, Grand Island, NY) were used to examine the gene of interest mRNA expression. The amplification cycling conditions were 95 °C for 2 min; 40 cycles of 95 °C for 10 s, 60 °C for 40 s. Control of the RT reactions was performed by omitting DNA template in the negative controls. The data were analysed by comparative C_T_ method and three replicates were performed. The primers for KLRG1 were forward 5′-CCAGACCGCTGGATGAAATATG-3′ and reverse 5′-CTGATTGTCCGTTATCACAAGGA-3′. The primers for BTK were forward 5′- TCTGAAGCGATCCCAACAGAA-3′ and reverse 5′-TGCACGGTCAAGAGAAACAGG-3′. The primers for CCR2 were forward 5′-CCACATCTCGTTCTCGGTTTATC-3′ and reverse 5′-CAGGGAGCACCGTAATCATAATC-3′. The primers for SCML4 were forward 5′-TCACTCCACGCCTATGAAGAT-3′ and reverse 5′-GGGTTTCCGCCCTCTTTTC-3′. The primers for ACTB (Beta-Actin) were forward 5′-CTGTCCCTGTATGCCTCTG-3′ and reverse 5′-ATGTCACGCACGATTTCC-3′.

ACTB was used as an internal control.

### Cell proliferation analysis

Control and the KLRG1-knockdown A549 cells (2 × 10^3^ cells per well) were plated in 48-well plates treated with E-Cadherin Antibody or IgG1 (Sinobiological, Beijing, China), and then cultured for 72 h. Cell proliferation was determined using the colorimetric Cell Counting Kit-8 (CCK-8, Dojindo Laboratories, Kumamoto, Japan). The absorbance was measured using Varioskan LUX (Thermo Fisher, Waltham, MA, USA) at a wavelength of 450 nm.

### Receiver operating characteristic (ROC) curve analysis

The diagnostic value of the expression levels of KLRG1, BTK, CCR2 and SCML4 in LUADs was studied by analyzing the expression data from 483 LUADs and 347 normal tissues. Specificity and sensitivity were plotted on the x- and y-axes, respectively. The area under curve (AUC) was calculated to assess the ability of the expression levels of KLRG1, BTK, CCR2 and SCML4 to predict the outcome of patients with LUAD.

### PPI network analysis and functional enrichment

Protein–protein (PPI) interactions network can visualize the patterns of molecular interactions and help to explain the mechanisms underlying phenotypes. PPI network analysis was performed using the online database STRING (https://string-db.org/) [18]. And the Gene Ontology (GO) enrichment and Kyoto Encyclopedia of Genes and Genomes (KEGG) pathway analysis were analysed by STRING.

### Statistical analysis

Assays were repeated in 2 or more biological experiments with each data point being the average of a minimum of 3 technical replicates. Statistical analysis was conducted using GraphPad Prism 6.0 (GraphPad Inc., La Jolla, California) or SPSS 20.0 software package (SPSS Inc., Chicago, Illinois). Group comparison was performed using two-tailed unpaired Student’s *t*-test. Prognostic factors were evaluated using univariate Cox regression analysis. The diagnostic and prognostic value of KLRG1, BTK, CCR2 and SCML4 expression in LUAD was judged using receiver operating characteristic (ROC) curves. Kaplan–Meier curves of OS and RFS were generated using GraphPad Prism. *P* values are derived from Log-rank (Mantel-Cox) test for all the survival analysis. *P* < 0.05 was considered statistically significant.

## Results

### The expression of KLRG1 is correlated with immunotherapy response and overall survival in LUAD patients

**Fig. 1:**
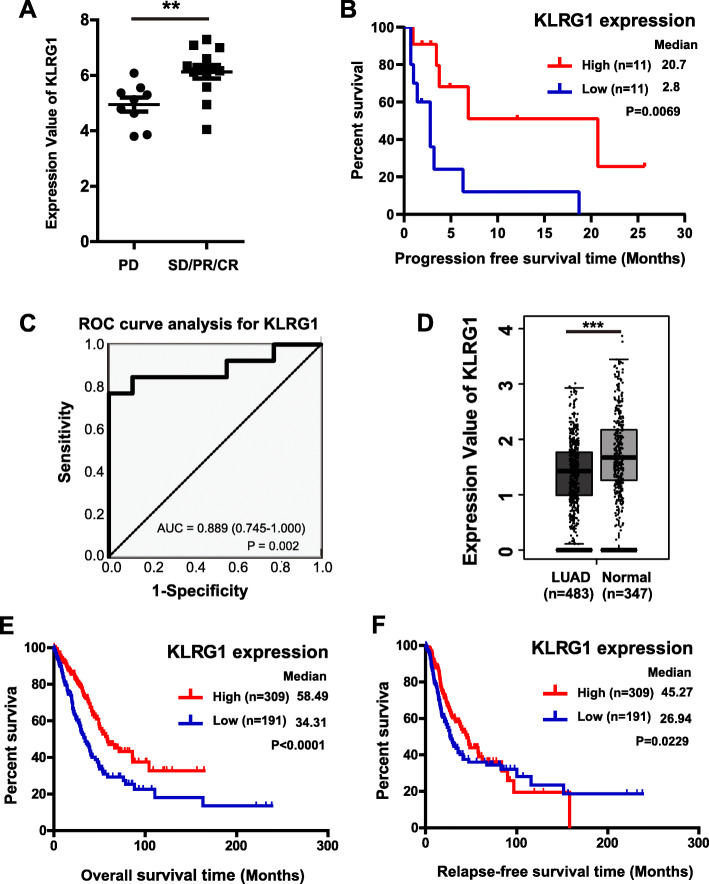
Influence of KLRG1 expression on LUAD survival and immunotherapy responses. **A** Expression of KLRG1 in 22 nonsquamous lung carcinoma patients with different response after PD-1 blockade. *P* values are derived from two-tailed unpaired Student’s *t*-test. **B** Kaplan–Meier curves of progression-free survival in the 22 nonsquamous lung carcinoma patients based on the mRNA expression of KLRG1. **C** Receiver operating characteristic curves for estimating the prognostic value of KLRG1 after PD-1 blockade. **D** Expression of KLRG1 in LUAD and normal lung tissues. The method for differential analysis is one-way ANOVA, using disease state (Tumor or Normal) as variable for calculating differential expression. **E** Kaplan–Meier curves of OS in LUAD based on the mRNA expression of KLRG1. **F** Kaplan–Meier curves of RFS in LUAD based on the mRNA expression of KLRG1. Data represent mean ± SD. **, *P* < 0.01, ***, *P* < 0.001

To find the potential biomarkers related to the development and immunotherapy response of lung adenocarcinoma. We analysed the data from 1 previous array (GSE93157) that compared gene expression profiles from 65 patients with melanoma, lung nonsquamous(*N* = 22), squamous cell lung or head and neck cancers who were treated with the approved PD1-targeting antibodies pembrolizumab or nivolumab [19]. The clinical and pathological characteristics of 22 lung nonsquamous patients were shown in Table 1. Among them, 59% (*N* = 13) of the patients had relatively good response including SD (stable disease), PR (partial response) and CR (complete response). And 41% (*N* = 9) of the patients showed PD (progressive disease). We analysed the differential expression genes between different responses. Then, we studied the differential survival genes in LUAD using data from GEPIA2. There were four genes had overlap in the two databases, which were KLRG1, CDK1, IL1R2, BTK and CCR2. Among them, we found that KLRG1 had the most significant correlation with the drug response after anti-PD1 treatment. KLRG1 expression was significantly higher in LUAD patients which showed better responses (SD, PR and CR) compared those patients with PD (Fig. 1a). The associations between the expression levels of KLRG1 and the clinicopathological characteristics of 22 lung nonsquamous patients were shown in Table 2. By conducting multivariate analysis, we found that, the expression level of KLRG1 was independent prognostic factor to predict the progression-free survival time (PFS) (*P* = 0.014) (Table 3). Furthermore, we investigated the association between KLRG1 expression and progression-free survival outcomes in lung nonsquamous patients after immunotherapy. The result showed that the high expression group had significantly better PFS compared to the low expression group (*P* = 0.0069) (Fig. 1b). Meanwhile, ROC curves and AUC analysis were performed to evaluate the prognostic performance. The results indicated that the performance of the KLRG1 had high sensitivity and specificity (Fig. 1c). These results suggested that KLRG1 maybe a potential biomarker to predict the immunotherapy response in LUAD patients.

**Table 1:** Clinical–pathologic characteristics of the 22 nonsquamous lung carcinoma patients evaluated in this study

	N (%)
N	22
Age, median (range)	58 (42–79)
Sex	
Male	7 (32%)
Female	15 (68%)
Previous lines	
0	4 (18%)
1	8 (37%)
2	6 (27%)
≥ 3	4(18%)
Biopsy	
Archival	10 (45%)
Baseline	12 (55%)
Drug response	
CR	1 (5%)
PR	5 (22%)
SD	7 (32%)
PD	9 (41%)
Smoking	
Current smoker	8 (36%)
Former smoker	11 (50%)
Never smoker	3 (14%)
ECOG	
0	5 (23%)
1	17 (77%)
Drug	
Nivolumab	14 (64%)
Pembrolizumab	8 (36%)
PFS, median	3.03
Lung cancer EGFR status	
EGFR mutated	1 (5%)
EGFR wild-type	21 (95%)
Lung cancer ALK status	
ALK rearranged	0 (0%)
ALK not rearranged	21 (95%)
NA	1 (5%)

*Abbreviations*: *CR* complete response, *PR* partial response, *SD* stable disease, *PD* progression disease, *PFS* progression-free survival, *ALK* anaplastic lymphoma kinase, *NA* not applicable

**Table 2:** Association of KLRG1 expression levels with clinicopathologic variables of 22 lung cancer patients

Outcome	KLRG1 expression		
	Low	High	*P* value
Age / year			0.838
<58	5	6	
≥ 58	6	5	
Gender			0.798
Male	5	9	
Female	6	2	
Drug			0.794
Nivolumab	7	7	
Pembrolizumab	4	4	
Smoking history / Category			0.123
Current smoker & Former smoker	1	2	
Never smoker	10	9	
EGFR			0.285
Mutated	1	0	
Wild-type	10	11	
ECOG			0.882
0	2	3	
1	9	8	
State			0.030
PD	8	1	
CR, PR, SD	3	10	

*P* values are derived from one-way analysis of variance. *Abbreviations*: *CR* complete response, *PR* partial response, *SD* stable disease, *PD* progression disease

**Table 3:** Statistically significant associations of KLRG1 expression and other clinicopathologic variables with progression-free survival

Outcome	PFS		
	HR	95%CI	*P* value
KLRG1 expression level (High vs. Low)	0.139	0.029–0.668	0.014
Drug (Nivolumab vs. Pembrolizumab)	8.781	1.332–57.878	0.024
Biopsy (Archival vs. Baseline)	0.248	0.094–1.842	0.248
Smoking history (Current smoker & Former smoker vs. Never smoker)	2.930	0.311–27.575	0.347
Gender (Male vs. Female)	0.263	0.048–1.447	0.125
Age (< 58 vs. ≥58)	0.417	0.110–1.579	0.198
ECOG (0 vs. 1)	0.480	0.093–2.480	0.381

*P* values are derived from Cox regression analysis

Meanwhile, using data from GEPIA2, we also found KLRG1 belonged to the most differential survival genes in LUAD. Using RNA-seq data in TCGA and GTEx projects, we compared the KLRG1 expression between cancerous and normal lung tissues. Results showed that LUAD tissues (*N* = 483) had significantly decreased KLRG1 expression compared to normal controls (*N* = 347, Fig. 1d). By generating Kaplan-Meier survival curves, we analyzed the association between KLRG1 expression and OS/RFS in patients with LUAD. The LUAD patients were divided into high/low KLRG1 expression group by using the best cutoff model. Results showed that the high KLRG1 expression group had significantly better overall survival (OS) (*P* < 0.01) and relapse-free survival (RFS) (*P* < 0.05) compared to the low KLRG1 expression group (Fig. 1e and f).

### BTK, CCR2 and SCML4 are positively co-expressed with KLRG1 in LUAD

**Fig. 2:**
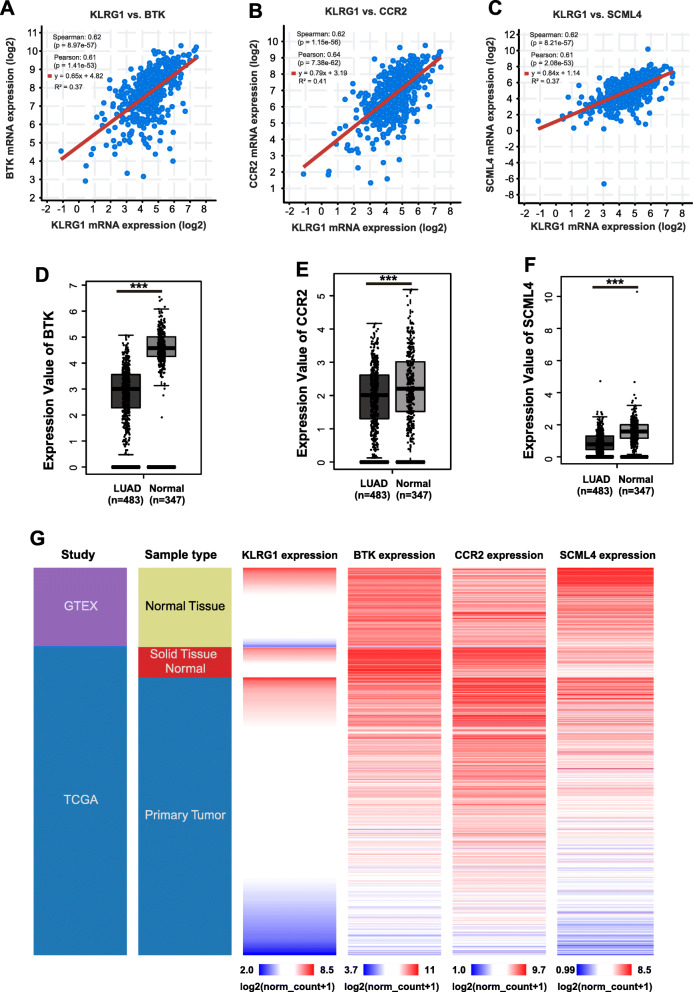
BTK, CCR2 and SCML4 were co-expressed with KLRG1 and downregulated in LUAD. **A**-**C** Regression analysis of the correlation between KLRG1 expression and BTK (**A**), CCR2 (**B**), SCML4 (**C**) expression, respectively. *P* values are derived from Spearman and Pearson correlation analysis. **D**-**F** Expression of BTK (**D**), CCR2 (**E**), SCML4 (**F**) in LUAD and normal lung tissues, respectively. *P* values are derived from one-way ANOVA. **G** Heatmap of KLRG1, BTK, CCR2 and SCML4 expression in LUAD patients and normal lung tissues. ***, *P* < 0.001

We next conducted the co-expression analysis in cBioPortal database, and found that BTK, CCR2 and SCML4 had a strong expression correlation with KLRG1 (Fig. 2a, b and c). Furthermore, BTK, CCR2 and SCML4 were among the top factors which expression can significantly influence the overall survival outcomes in LUAD. To further investigate the role of BTK, CCR2 and SCML4 in LUAD, we compared the expression of BTK, CCR2 and SCML4 between cancerous and normal lung tissues using the RNA-seq data in TCGA and GTEx projects, respectively. In the data cohort, RNA-seq was performed in 483 LUAD tissues and 347 normal tissues. The plots chart showed that BTK, CCR2 and SCML4 was significantly downregulated in LUAD tissues compared with the normal controls (Fig. 2d, e and f). Heatmap also showed that KLRG1, BTK, CCR2 and SCML4 expressions were significantly higher in normal tissues than in LUAD tissues (Fig. 2g).

### Knockdown of KLRG1 promotes the proliferation of A549 and H1299 tumor cells

**Fig. 3:**
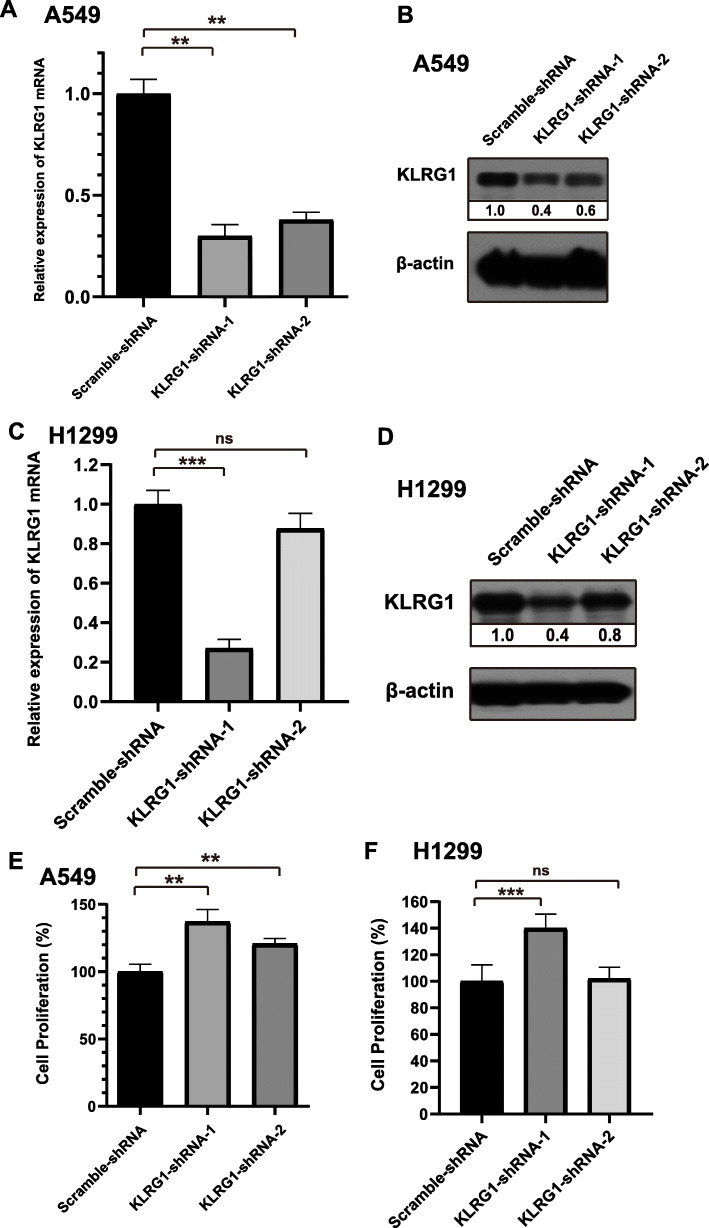
Knockdown of KLRG1 in A549 and H1299 lung tumor cells. **A**-**B** The KLRG1 knockdown efficiency in A549 was evaluated by qRT-PCR, *n* = 3 for each group (**A**) and Western blot (**B**). **C**-**D** The KLRG1 knockdown efficiency in H1299 was evaluated by qRT-PCR, *n* = 3 for each group (**C**) and Western blot (**D**). The full-length gels are presented in Supplementary Figure 1 and Figure 2. **E**-**F** The effect of KLRG1-knockdown on the proliferation of A549 (**E**) and H1299 (**F**) tumor cells, *n* = 4 for each group. *P* values are derived from two-tailed unpaired Student’s *t*-test. Data represent mean ± SD. **, *P* < 0.01, ***, *P* < 0.001, ns, no significance

To further understand the role of KLRG1 in the development of LUAD, we generated stable KLRG1 knockdown cells by transfection with KLRG1-specific short hairpin RNAs (shRNAs). The knockdown efficiency of KLRG1 in A549 and H1299 cells were confirmed by both qRT-PCR and Western blotting (Fig. 3a-d). Moreover, we investigated the effect of KLRG1 knockdown on the proliferation of A549 and H1299 cells. The results showed that knockdown of KLRG1 enhanced the proliferation of A549 (Fig. 3e) and H1299 (Fig. 3f) tumor cells. The shRNA2 did not influence the proliferation of H1299 tumor cells because of the low knockdown efficiency in H1299 tumor cells. These results indicated that KLRG1 may promote the development of LUAD.

### ROC analysis of the BTK, CCR2, SCML4 and KLRG1 expression in patients with LUAD

**Fig. 4:**
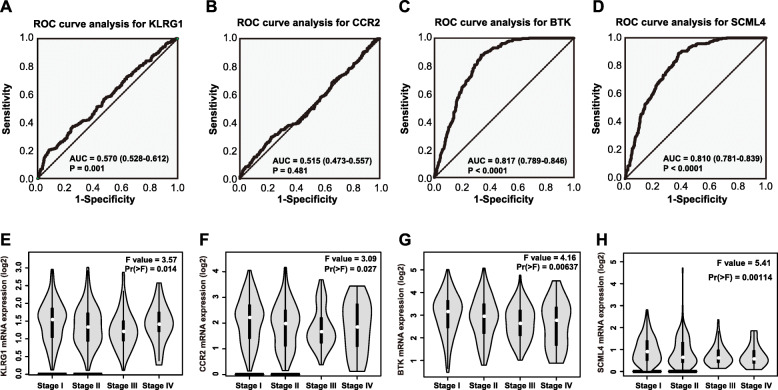
The diagnostic value of KLRG1, CCR2, BTK and SCML4 expression in LUAD. **A**-**D** Receiver operating characteristic curves for estimating the diagnostic value of KLRG1 (**A**), CCR2 (**B**), BTK (**C**), and SCML4 (**D**). **E**-**H** the KLRG1 (**E**), CCR2 (**F**), BTK (**G**) and SCML4 (**H**) expressions in different pathological stages of LUAD

Since the four factors were downregulated in LUAD samples compared with controls, we next explored whether the four factors may serve as potential diagnostic biomarkers in LUAD. Diagnostic ROC curves and AUC analysis were performed to evaluate the diagnostic performance. The results indicated that the performance of the KLRG1 [AUC, 0.570; 95% CI (confidence interval),0.528–0.612] (Fig. 4a) and CCR2 (AUC, 0.515; 95% CI, 0.473–0.557) (Fig. 4b) were not satisfactory, but BTK (AUC, 0.871; 95% CI, 0.789–0.846) (Fig. 4c) and SCML4 (AUC, 0.810; 95% CI, 0.781–0.839) (Fig. 4d) had high sensitivity and specificity. It suggested that the BTK and SCML4 may have huge value in the auxiliary diagnosis of LUAD patients.

### Association between KLRG1, BTK, CCR2, SCML4 expression and the demographic and clinicopathological parameters of patients with LUAD

Then, we investigated the associations between the expression levels of KLRG1, BTK, CCR2, SCML4 and the clinicopathological characteristics of 499 patients with LUAD. The results showed that the expression level of CCR2 was associated with age (*P* = 0.040; Table 4). Meanwhile, the expression level of CCR2 and SCML4 was associated with gender (*P* = 0.029; *P* = 0.006, respectively; Table 1). Notably, the expression levels of KLRG1, BTK, CCR2, SCML4 were all associated with neoplasm disease stage (American Joint Committee on Cancer Code) (*P* = 0.001; *P* = 0.022; *P* = 0.004; *P* = 0.014, respectively; Table 4). And a lower expression levels of KLRG1, BTK, CCR2, SCML4 were all associated with advanced neoplasm disease stage (Fig. 4e-h). By conducting multivariate analysis, we found that, in addition to neoplasm disease stage and diagnosis age, the expression levels of KLRG1, BTK, CCR2 and SCML4 were all independent prognostic factor for OS (hazard ratio, HR = 1.658 and *P* = 0.002 for KLRG1; HR = 1.889 and *P* < 0.001 for BTK; HR = 1.922 and *P* < 0.001 for CCR2; HR = 1.638 and *P* = 0.001 for SCML4) in LUAD patients (Table 5).

**Table 4:** The association between KLRG1, BTK, CCR2, SCML4 expression and the demographic and clinicopathological parameters

Outcome ^a^	KLRG1 expression			BTK expression			CCR2 expression			SCML4 expression		
	Low	High	*P* value	Low	High	*P* value	Low	High	*P* value	Low	High	*P* value
Age / year			0.250			0.082			0.040			0.172
<65	145	73		132	86		134	84		96	122	
≥ 65	160	111		142	129		136	135		97	174	
Gender			0.710			0.156			0.029			0.006
Male	146	84		137	93		140	90		105	125	
Female	165	104		141	128		134	135		92	177	
Mutations / number			0.615			0.067			0692			0.103
≤ 230	92	50		90	54		77	67		56	88	
>230	52	25		40	35		39	36		37	38	
Smoking history / Category^b^			0.628			0.112			0.280			0.937
1	40	31		32	39		32	39		26	45	
2, 3, 4, 5	262	152		239	175		235	179		165	249	
Neoplasm Disease Stage			0.001			0.022			0.004			0.014
I, II	224	162		202	184		196	190		141	245	
III, IV, V	82	23		72	33		72	33		54	51	

*P* values are derived from one-way analysis of variance

^a^ A total of 499 samples were analyzed. Age: *N* = 489, NA = 10; Stage: *N* = 491, NA = 8; Mutations: *N* = 219, NA = 280; Smoking History: *N* = 485, NA = 14. N: Number; NA: Not Applicable

^b^ Smoking history: 1: lifelong non-smoker; 2: current smoker; 3. Current reformed smoker (for> 15 years); 4. Current reformed smoker (for≤15 years); 5. Current reformed smoker (duration not specified)

**Table 5:** Statistically significant associations of KLRG1, BTK, CCR2 and SCML4 expressions with overall survival in LUAD patients

Outcome	OS		
	HR	95% CI	*P* value
KLRG1 expression level (high vs. low)	1.658	1.202–2.288	0.002
BTK expression level (high vs. low)	1.889	1.381–2.585	< 0.001
CCR2 expression level (high vs. low)	1.922	1.404–2.631	< 0.001
SCML4 expression level (high vs. low)	1.638	1.225–2.191	0.001
Diagnosis Age < 65 vs. ≥ 65	3.553	1.122–11.253	0.031
Neoplasm Disease Stage I, II vs. III, IV, V	1.788	1.391–2.298	< 0.001
Mutation Count < 230 vs. ≥ 230	1.001	0.850–1.178	0.995
Gender Female vs. Male	1.043	0.779–1.396	0.779
Smoking History Category^a^ 1 vs. 2, 3, 4, 5	0.966	0.681–1.371	0.847

*P* values are derived from Cox regression analysis

^a^Smoking history: 1: lifelong non-smoker; 2: current smoker; 3. Current reformed smoker (for> 15 years); 4. Current reformed smoker (for≤15 years); 5. Current reformed smoker (duration not specified)

### Prognostic value of the BTK, CCR2, SCML4 and KLRG1 in patients with LUAD

**Fig. 5:**
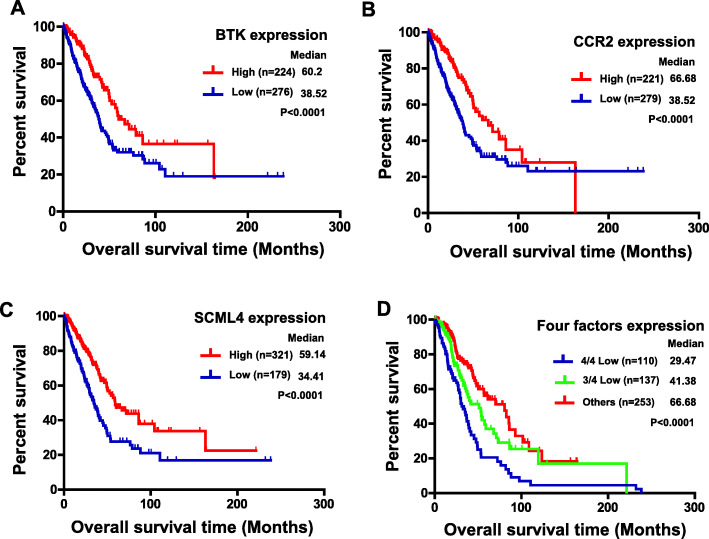
Kaplan-Meier survival curves for assessing the prognostic value of KLRG1, BTK, CCR2 and SCML4. Kaplan–Meier analysis for the OS of LUAD patients according to distinct BTK (**A**), CCR2 (**B**) and SCML4 (**C**) expression level. **D** Kaplan–Meier analysis for the OS of LUAD patients according to the expression of the four factors

To examine the association between BTK, CCR2 and SCML4 expression and survival outcomes in LUAD, respectively, we extracted the survival data in TCGA database. The LUAD patients were divided into high/low expression group by using the best cutoff model. For all the three genes, results showed that the high expression groups had significantly better OS compared to the low expression groups, respectively (*P* < 0.0001 for all, Fig. 5a, b and c). Furthermore, we combined the expression of the four factors (KLRG1, BTK, CCR2 and SCML4), the patients were divided into three groups. Group 1 included 110 patients which had all low expression of the four factors. Group 2 included 137 patients with three factors low expression among the four. Group 3 included the rest of the patients (*N* = 253). Strikingly, the patients which had all low expression of the four factors showed the worst overall survival (*P* < 0.0001, median survival time = 29.5, 41.4, 66.7, respectively, Fig. 5d). These results indicated that the expression of the four factors could be powerful biomarkers to predict the LUAD patient survival.

### Protein-protein interaction network analysis

**Fig. 6:**
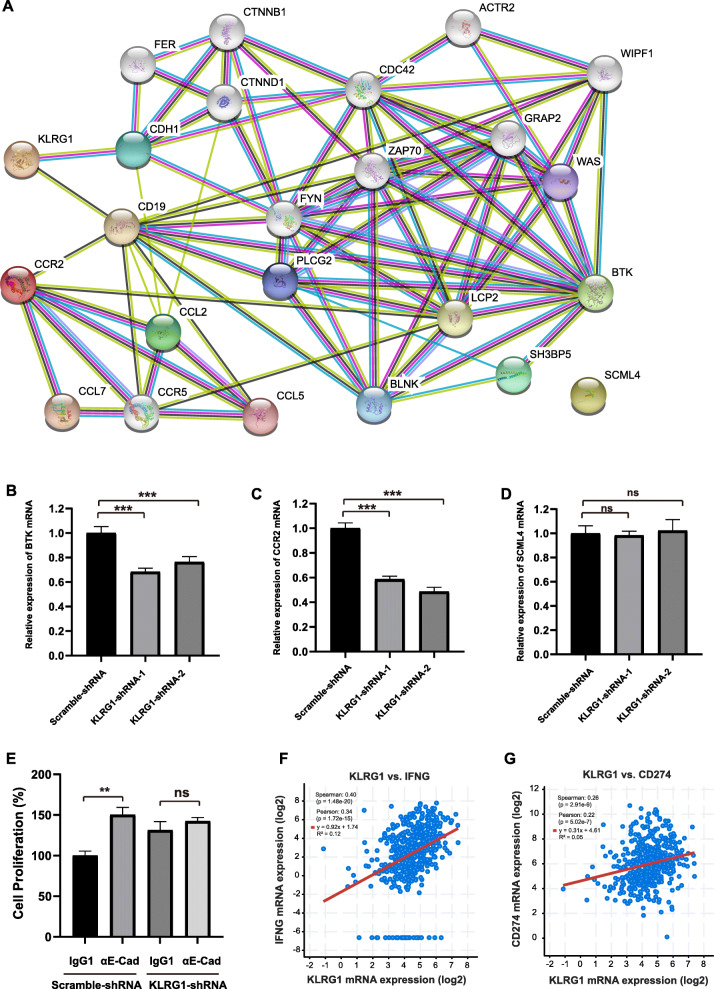
The interaction between KLRG1, BTK and CCR2 influences the proliferation of lung tumor cells. **A** Protein-protein interaction analysis of KLRG1, BTK, CCR2, SCML4 and their-related genes. Each node represents a different gene. Each line represents a connection between two different genes. **B**-**D** The expression of BTK (**B**), CCR2 (**C**) and SCML4 (**D**) in KLRG1-knockdown A549 tumor cells were evaluated by qRT-PCR, *n* = 3 for each group, *P* values are derived from two-tailed unpaired Student’s *t*-test. **E** The effect of E-Cadherin antibody or IgG1 treatment on the proliferation of A549 tumor cells treated with scramble or KLRG1 shRNA, *n* = 4 for each group, *P* values are derived from two-tailed unpaired Student’s *t*-test. **F**-**G** Regression analysis of the correlation between KLRG1 and IFNG (**F**) or CD274 (**G**), *P* values are derived from Spearman and Pearson correlation analysis. Data represent mean ± SD. **, *P* < 0.01, ***, *P* < 0.001, ns, no significance

In order to study how these factors were involved in the development of LUAD, STRING was performed to construct the protein-protein interactions among KLRG1, BTK, CCR2 and SCML4. The number of nodes was 24 and number of edges was 80. The PPI enrichment *P*-value was 7.13e-10. In the PPI network, KLRG1, BTK and CCR2 had close connections through CD19 (CD19 molecule), CDH1 (Cadherin 1), FYN (Src family tyrosine kinase), PLCG2 (Phospholipase C gamma 2) and LCP2 (Lymphocyte cytosolic protein 2) (Fig. 6a). Due to few studies based on SCML4, it showed an independent relationship in the PPI network. Furthermore, we did functional analysis based on the PPI network. The top processes and pathways were showed in Table 6. To further investigate the interaction of the four factors, we analyzed the expression levels of BTK, CCR2 and SCLM4 in KLRG1 knockdown A549 tumor cells. The results showed that knockdown of KLRG1 also reduced the expression of BTK and CCR2 in A549 tumor cells (Fig. 6b and c). On the other hand, the expression of SCLM4 did not show significant changes (Fig. 6d). E-cadherin was reported as the ligand of KLRG1. And the treatment of E-Cadherin antibody enhanced the proliferation of A549 tumor cells (Fig. 6e). The tumor promotion effect was eliminated in KLRG1 knockdown A549 cells (Fig. 6e). This indicated that KLRG1 may influence the proliferation of tumor cells through the biding of E-cadherin. As KLRG1 was abundantly expressed on the surface of T cells and NK cells, we speculated that the expression of KLRG1 on tumor cells would competitively bind E-cadherin in tumor microenvironment which would promote the activity of T cells and NK cells. We conducted the co-expression analysis in cBioPortal database, and found that IFNG and CD274 had a good expression correlation with KLRG1 (Fig. 6f and g). The results indicated that the high expression of KLRG1 on tumor cells may induce the activation of immune cells. Altogether, these results revealed that KLRG1, BTK and CCR2 may interact through cell surface receptor signaling pathway to influence the proliferation of tumor cells or affect the immune response indirectly.

**Table 6:** Functional and pathway enrichment analysis

Category	ID	Term Description	Observed gene count	Background gene count	***P***-value	Included Gene
Biological Process (GO)	GO:0007166	Cell Surface Receptor Signaling Pathway	31	2198	2.11E-23	BTK,CCR2,KLRG1
	GO:0002376	Immune System Process	28	2370	2.94E-18	BTK,CCR2,KLRG1
	GO:0007165	Signal Transduction	32	4738	1.10E-15	BTK,CCR2,KLRG1
	GO:0006955	Immune Response	23	1560	1.11E-15	BTK,CCR2,KLRG1
	GO:0051716	Cellular Response to Stimulus	33	6212	7.71E-14	BTK,CCR2,KLRG1
	GO:0006952	Defense Response	18	1234	1.82E-11	BTK,CCR2,KLRG1
	GO:0045087	Innate Immune Response	14	676	1.68E-10	BTK,KLRG1
	GO:0006950	Response to Stress	22	3267	1.95E-08	BTK,CCR2,KLRG1
	GO:0050794	Regulation of Cellular Process	34	10,484	2.12E-08	BTK,CCR2,KLRG1,SCML4
	GO:0006954	Inflammatory Response	9	482	2.51E-06	BTK,CCR2,KLRG1
Molecular Function (GO)	GO:0005515	Protein Binding	29	6605	1.32E-07	BTK,CCR2
	GO:0005102	Signaling Receptor Binding	15	1513	7.57E-07	BTK,CCR2
	GO:0005488	Binding	31	11,878	0.001	BTK,CCR2,KLRG1
	GO:0042802	Identical Protein Binding	9	1754	0.0128	BTK,CCR2
Cellular Component (GO)	GO:0071944	Cell Periphery	23	5254	2.02E-05	BTK,CCR2,KLRG1
	GO:0005886	Plasma Membrane	22	5159	5.39E-05	BTK,CCR2,KLRG1
	GO:0005623	Cell	34	16,271	0.011	BTK,CCR2,KLRG1,SCML4
	GO:0016020	Membrane	23	8420	0.0169	BTK,CCR2,KLRG1
	GO:0005622	Intracellular	31	14,286	0.037	BTK,CCR2,KLRG1,SCML4
KEGG Pathways	hsa04662	B Cell Receptor Signaling Pathway	8	71	3.96E-11	BTK
	hsa04062	Chemokine Signaling Pathway	8	181	2.36E-08	CCR2
	hsa04664	Fc Epsilon Ri Signaling Pathway	6	67	5.25E-08	BTK
	hsa05340	Primary Immunodeficiency	5	37	1.53E-07	BTK
Reactome Pathways	HSA-168256	Immune System	27	1925	4.00E-19	BTK,CCR2,KLRG1
	HSA-1280218	Adaptive Immune System	16	733	8.03E-13	BTK,KLRG1
	HSA-168249	Innate Immune System	17	1012	5.02E-12	BTK,CCR2
	HSA-162582	Signal Transduction	22	2605	1.53E-10	BTK,CCR2
UniProt Keywords	KW-0391	Immunity	8	507	5.56E-05	BTK,KLRG1
	KW-1003	Cell Membrane	16	3208	0.00064	BTK,CCR2,KLRG1
	KW-0597	Phosphoprotein	25	8066	0.0018	BTK,CCR2,SCML4
	KW-0399	Innate Immunity	4	309	0.0141	BTK,KLRG1

*P* values are derived from false discovery rate corrections

## Discussion

In the past few decades, experts have explored the mechanisms of lung adenocarcinoma formation and development through extensive basic and clinical research. The treatment of LUAD also has made huge progress. However, it is an urgent need to develop biomarkers to predict and monitor the response and clinical benefit of immunotherapy in LUAD patients. In this study, through the analyze of the data from the Gene Expression Omnibus, the Cancer Genome Atlas and the Genotype-Tissue Expression, we found that the expression of KLRG1, BTK, CCR2 and SCML4 was significantly downregulated in LUAD tissues compared to normal controls. And the expression of these four factors significantly predicted the overall survival time in patients with LUAD. Furthermore, low expression of KLRG1 also correlated with poor responses in LUAD patients after immune-checkpoint inhibitor treatment.

Killer-cell lectin like receptor G1 (KLRG1) is expressed on NK cells and antigen-experienced T cells and has been assumed to be a marker of senescence [20]. Despite the extensive use of KLRG1 as a marker of differentiation, KLRG1 possesses an immune receptor tyrosine-based inhibitory motif (ITIM) in its cytoplasmic domain, suggesting that it may play an inhibitory role in human NK cells and T cells [21, 22]. KLRG1 has been little studied in human tumor samples. There is only one paper published in *Oncotarget* which investigated the expression of KLRG1 on tumor cells. In that study, it showed that KLRG1 expression is upregulated in human tumor samples after a variety of therapies, including radiation, endocrine therapy, chemotherapy, and immunotherapy [23]. And we are the first study to focus on the direct role of KLRG1 on tumor cells. In our study, we found that low expression of KLRG1 also correlated with poor responses in LUAD patients after immune-checkpoint inhibitor treatment. Direct assessment of PD-L1 expression on tumor cells is a logical biomarker for the prediction of treatment response to anti-PD-1 or anti-PD-L1 therapies. However, patients show PD-L1-negative by immunohistochemistry can still achieve clinical benefit with anti-PD-1 or anti-PD-L1 therapies. KLRG1-mediated inhibition of NK cell function reveals that KLRG1/ligand interactions inhibit the cytolytic activity of polyclonal human NK cells by interfering with both degranulation and interferon γ (IFNγ) release [24]. And blocking KLRG1 signaling during TCR activation using antibodies against its ligand, E-cadherin, enhances proliferative activity of T cells [25]. Meanwhile, some study already described that immune gene signatures, especially those induced by IFNγ, might be robust biomarkers for predicting clinical benefit to anti-PD-1 or anti-PD-L1 therapies [6]. Consistent with these studies, it led us to propose that the expression of KLRG1 on LUAD tumor cells will competitively bind to its ligand E-cadherin with T cells or NK cells. The decreased level of E-cadherin in the tumor microenvironment will reduce the inhibitory role of KLRG1 on T cells and NK cells, which leads to high release of degranulation and interferon γ. Consequently, the anti-tumor effect of the immune system is strengthened. On the other hand, the expression of KLRG1 was directly related to the overall survival and progress free survival of LUAD patients. So, KLRG1 may also play a directly role to the tumor differentiation and progression. Our results also indicated that knockdown of KLRG1 promoted the proliferation of A549 lung cancer cells. And E-cadherin maybe the ligand of KLRG1 on tumor cells. How KLRG1 influences tumor cells proliferation is an interesting question which deserves further study. On the other hand, the expression of KLRG1 is not very high in lung tumor tissues which is indeed a limitation as a biomarker. So, we need sensitive methods, such as PCR, to detect the expression of KLRG1 in tumor tissues.

Bruton’s tyrosine kinase (BTK), a Tec family non-receptor protein kinase, plays a crucial role in B-cell activation, proliferation, maturation, differentiation, and survival [26]. BTK has emerged as a novel molecular target in some B-cell leukemias and lymphomas where it is commonly overexpressed [27]. A Phase 1b/2 Study investigated the efficacy of the Bruton tyrosine kinase inhibitor Ibrutinib and the PD-L1 inhibitor Durvalumab in patients with pretreated solid tumors which includes 28 non-small cell lung cancer patients. The results indicated that the combination of ibrutinib and durvalumab did not show meaningful activity in any of the tumor types studied, and, therefore, recruitment was stopped due to lack of efficacy [28]. These results indicated that BTK may play different roles in LUAD compared with B-cell malignancies. The expression of BTK in different tumor types also implies this point. BTK is overexpressed in Diffuse Large B-cell Lymphoma (DLBCL) than normal tissues, but has a lower expression in LUAD and LUSC than normal tissues. So, it is really an interesting question that how BTK influences the development of lung cancer.

The CCL2/CCR2 signaling axis is first characterized as a chemotactic molecule with physiological regulating roles in inflammation. And the CCL2/CCR2 axis has generated increasing interest in recent years due to its association with the progression of cancer. On the other hand, CCL2/CCR2 has been shown to exert both pro- and anti-tumor effects [29]. CCL2 may also act to attract antitumor immune cells and is required for efficient immunosurveillance, implying that the inhibition of CCL2 may promote neocarcinogenesis as well as the development of metastases [30–32]. In our study, the expression of CCR2 was positively correlated with patients’ survival which indicated that CCL2/CCR2 axis may play anti-tumor effects in LUAD. Very little research is focused on SCML4 (Scm Polycomb Group Protein Like 4). But a study shown that SCML4 makes functional contributions to processes critical for atherosclerosis (endothelial cell activation and survival, inflammation, and adhesion) and decreased expression of SCML4 exacerbates endothelial dysfunction and vascular remodeling in a rat model [33].

In our study, we found that KLRG1, BTK, CCR2 and SCML4 were co-expressed in LUAD patients. Knockdown of KLRG1 enhanced the proliferation of A549 lung tumor cells. And low expression of the four factors associated with unfavorable overall survival in LUAD patients. Combined the analysis of protein-protein interaction, it implies that KLRG1, BTK, CCR2 and SCML4 may influence the LUAD development through immune system process. The detailed mechanisms of these four factors involved in LUAD development are intriguing questions needed to be investigated.

## Conclusions

Altogether, the results presented here indicate that KLRG1, BTK, CCR2 and SCML4 play important roles in the development of lung adenocarcinoma, and their expression can be effective biomarkers to predict LUAD patients’ survival. Notably, the expression of KLRG1 is also a good biomarker to predict the treatment response of immune-checkpoint inhibitors.

## Supplementary Information

**Additional file 1: Supplementary Figure 1.** Uncropped western blots used in Fig. 3b. The figure shows all original uncropped blots. The western blots of KLRG1-shRNA3-5 were not shown in the manuscript and Fig. 3b, because they did not shown significant knockdown efficacy. Blots were cropped where indicated by the red lines. **Supplementary Figure 2.** Uncropped western blots used in Fig. 3e. The figure shows all original uncropped blots. Blots were cropped where indicated by the red lines.

## Data Availability

All data are included in this article.
