# Supplementary material for: Functions and clinical significance of KLRG1 in the development of lung adenocarcinoma and immunotherapy
Source: BMC Cancer. 2021 Jun 29;21:752. doi: 10.1186/s12885-021-08510-3 (PMC8243757; doi:10.1186/s12885-021-08510-3)
Supplement: Supplementary file 1 — Additional file 1: Supplementary Figure 1. Uncropped western blots used in Fig. 3b. The figure shows all original uncropped blots. The western blots of KLRG1-shRNA3-5 were not shown in the manuscript and Fig. 3b, because they did not shown significant knockdown efficacy. Blots were cropped where indicated by the red lines. Supplementary Figure 2. Uncropped western blots used in Fig. 3e. The figure shows all original uncropped blots. Blots were cropped where indicated by the red lines. [file 12885_2021_8510_MOESM1_ESM.pptx]

## Slide 1
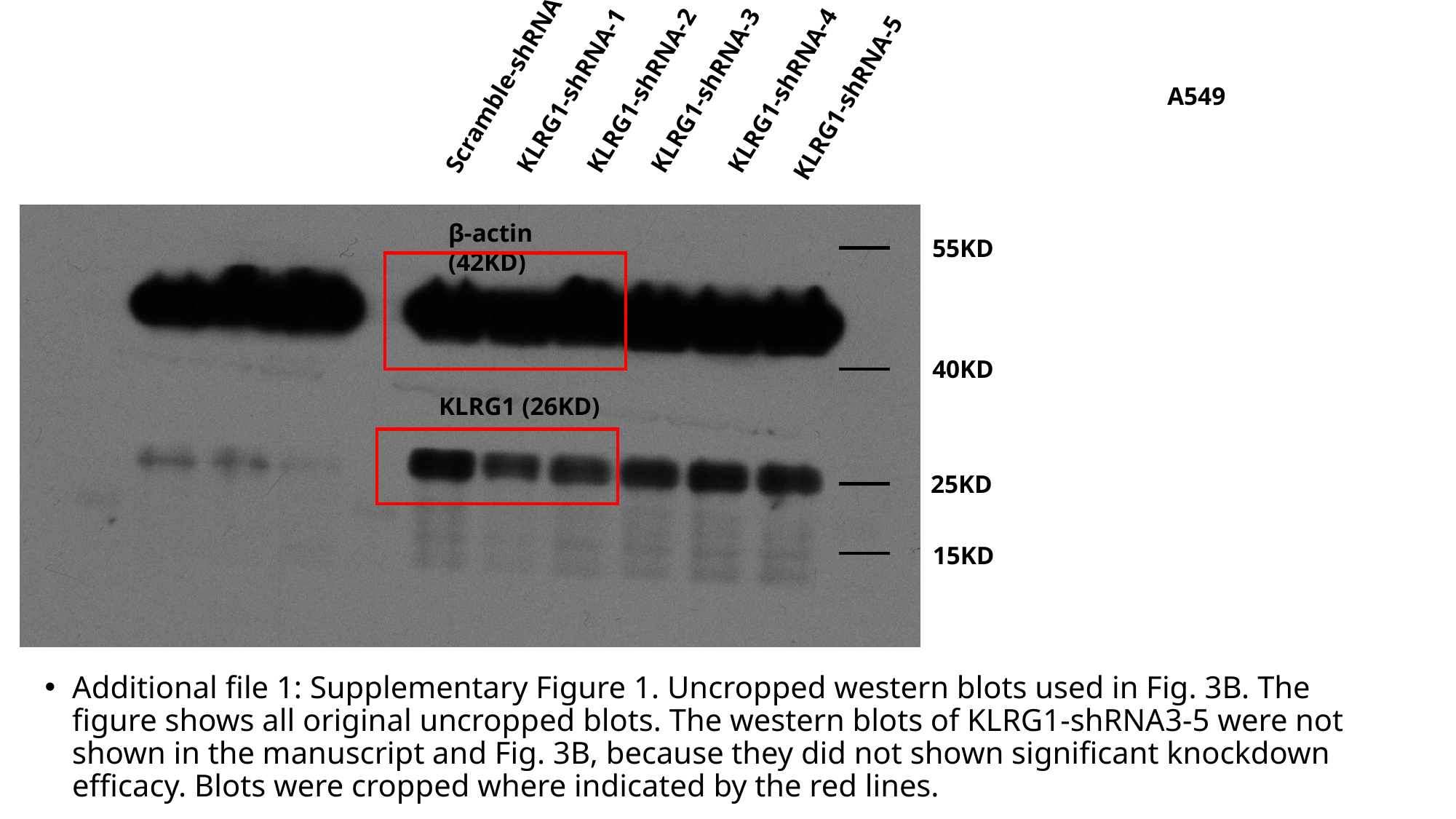

KLRG1-shRNA-1
KLRG1-shRNA-2
KLRG1-shRNA-3
KLRG1-shRNA-4
Scramble-shRNA
KLRG1-shRNA-5
β-actin (42KD)
KLRG1 (26KD)
A549
55KD
40KD
25KD
15KD
Additional file 1: Supplementary Figure 1. Uncropped western blots used in Fig. 3B. The figure shows all original uncropped blots. The western blots of KLRG1-shRNA3-5 were not shown in the manuscript and Fig. 3B, because they did not shown significant knockdown efficacy. Blots were cropped where indicated by the red lines.

## Slide 2
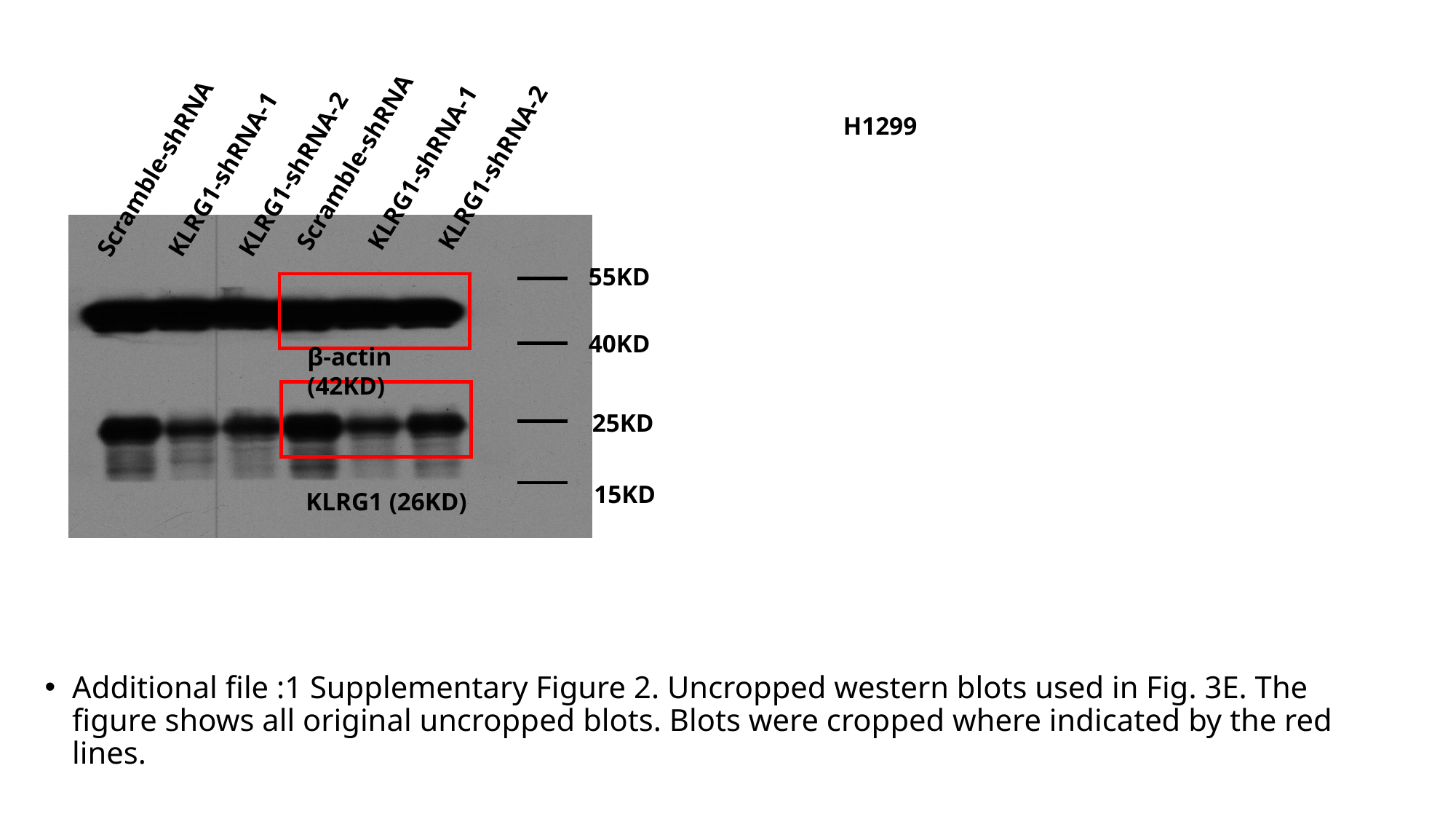

KLRG1-shRNA-1
KLRG1-shRNA-2
Scramble-shRNA
KLRG1-shRNA-1
KLRG1-shRNA-2
Scramble-shRNA
H1299
55KD
40KD
β-actin (42KD)
25KD
15KD
KLRG1 (26KD)
Additional file :1 Supplementary Figure 2. Uncropped western blots used in Fig. 3E. The figure shows all original uncropped blots. Blots were cropped where indicated by the red lines.
